# Development of Apical Blebbing in the Boar Epididymis

**DOI:** 10.1371/journal.pone.0126848

**Published:** 2015-05-21

**Authors:** Jennifer Hughes, Trish Berger

**Affiliations:** Department of Animal Science, University of California Davis, Davis, California, United States of America; Colorado State University, UNITED STATES

## Abstract

Microvesicles are of increasing interest in biology as part of normal function of numerous systems; from the immune system (T cell activation) to implantation of the embryo (invasion of the trophoblasts) and sperm maturation (protein transfer in the epididymis). Yet, the mechanisms involved in the appearance of apical blebbing from healthy cells as part of their normal function remain understudied. Microvesicles are produced via one of two pathways: exocytosis or apical blebbing also termed ectocytosis. This work quantifies the histological appearance of apical blebbing in the porcine epididymis during development and examines the role of endogenous estrogens in regulating this blebbing. Apical blebbing appears at puberty and increases in a linear manner into sexual maturity suggesting that this blebbing is a mature phenotype. Endogenous estrogen levels were reduced with an aromatase inhibitor but such a reduction did not affect apical blebbing in treated animals compared with their vehicle-treated littermates. Epididymal production of apical blebs is a secretion mechanism of functionally mature principal cells regulated by factors other than estradiol.

## Introduction

Microvesicles impact a variety of biological systems [1–5]. These systems include normal physiology as well as pathological states. Release of functional proteins, mRNAs and miRNAs from cells via microvesicles has generated interest in the mechanisms of cellular secretion of vesicles via apical blebbing, one of two pathways of microvesicle secretion [3–7]. Apical blebs are frequently observed in epithelial cells as part of the processes of secretion and apoptosis and as a byproduct of the processes of cytokinesis, cell movement and cell growth [8]. This non-classical secretion mechanism variously termed apical blebbing, apical budding or ectocytosis, is typically studied in cells undergoing this process *in vitro* [9,10]. However, the regulation of apical blebbing in an *in vivo* healthy environment has yet to be elucidated.

The porcine epididymis, an organ of the male reproductive tract, lends itself to the study of the development of apical blebbing, since it is an accessible organ with development spaced over a few months and apical blebbing is present in sexually mature males [11–13]. The epididymis is highly dependent on products of the testis for normal function, e.g. androgens, estrogens, sperm and non-hormonal testicular factors [14–17]. Manipulation of the regulatory environment of the epididymis is relatively straightforward, providing an *in vivo* system to evaluate the regulation of apical blebbing.

The epididymis functions to mature spermatozoa as they transit this excurrent duct. Transfer of epididymally synthesized proteins to the sperm plasma membrane occurs during sperm passage through the epididymis [18–21]. This transfer is mediated by microvesicles, termed epididymosomes, which are a product of apical blebbing of the epididymal epithelium [3]. Proteins present in these vesicles do not contain a signal sequence that would direct them into the ER/Golgi complex. Therefore, these proteins are translated on ribosomes in the cytosol and are delivered to the apical membrane for export [6,22].

Secretion via apical blebbing appears to be a metabolically demanding process as blebbing includes loss of plasma membrane and cytosol to the luminal environment. Information about the timing of the initiation of the blebbing process should offer insight on its regulation and function. Our first aim was to evaluate when apical blebbing develops in the porcine epididymis. Our second aim was to evaluate the contribution of endogenous estrogens to regulation of apical blebbing in the epididymis. Both estrogen and androgen pathways have been implicated in regulation of apical blebbing in the rat coagulation gland [23], and estrogen levels in boars are high compared with males from many other species. Inhibition of estrogen synthesis by the aromatase inhibitor, letrozole, was used to explore the role of endogenous estrogens in the regulation of apical blebbing [24–27].

## Materials and Methods

### Ethics Statement

Animal use and care was approved by the UC Davis Institutional Animal Care and Use Committee (protocols #16308 & #13398). Animals were housed with their littermates, under natural light in concrete floored pens in a specific pathogen free, AAALAC approved facility. Feed and water were available *ad libitum* through 20 weeks of age and a maintenance ration provided thereafter. Animals were monitored daily.

### Development of Apical Blebbing

Apical blebbing was confirmed in mature tissues (40 weeks of age) and no blebbing was evident in young animals (4 weeks of age) in our preliminary work, leading to the hypothesis that apical blebbing is a marker for epididymal maturity. Epididymides from boars derived from stock provided by PIC USA (Franklin, KY, USA) and maintained with semen provided by Genus plc (Hendersonville, TN, USA) housed at the UCDavis Swine Center were evaluated at 11, 16, 20 and 40 weeks of age (five animals at each age.) The 15 boars examined at 16, 20 and 40 weeks of age were littermates (five different litters) with each litter represented once at each age. Littermates were randomly assigned to the three ages for tissue collection. Boars were transported to the university abattoir and stunned via electrocution prior to exsanguination, an approved method of euthanasia for pigs [28]. Body weights at tissue collection were: 33.5 kg SEM = 4.9 at 11 weeks, 65.5 kg SEM = 3.9 at 16 weeks, 103 kg SEM = 4 at 20 weeks and 158 kg SEM = 5 at 40 weeks.

### Estrogenic Regulation of Apical Blebbing

**Fig 1:**
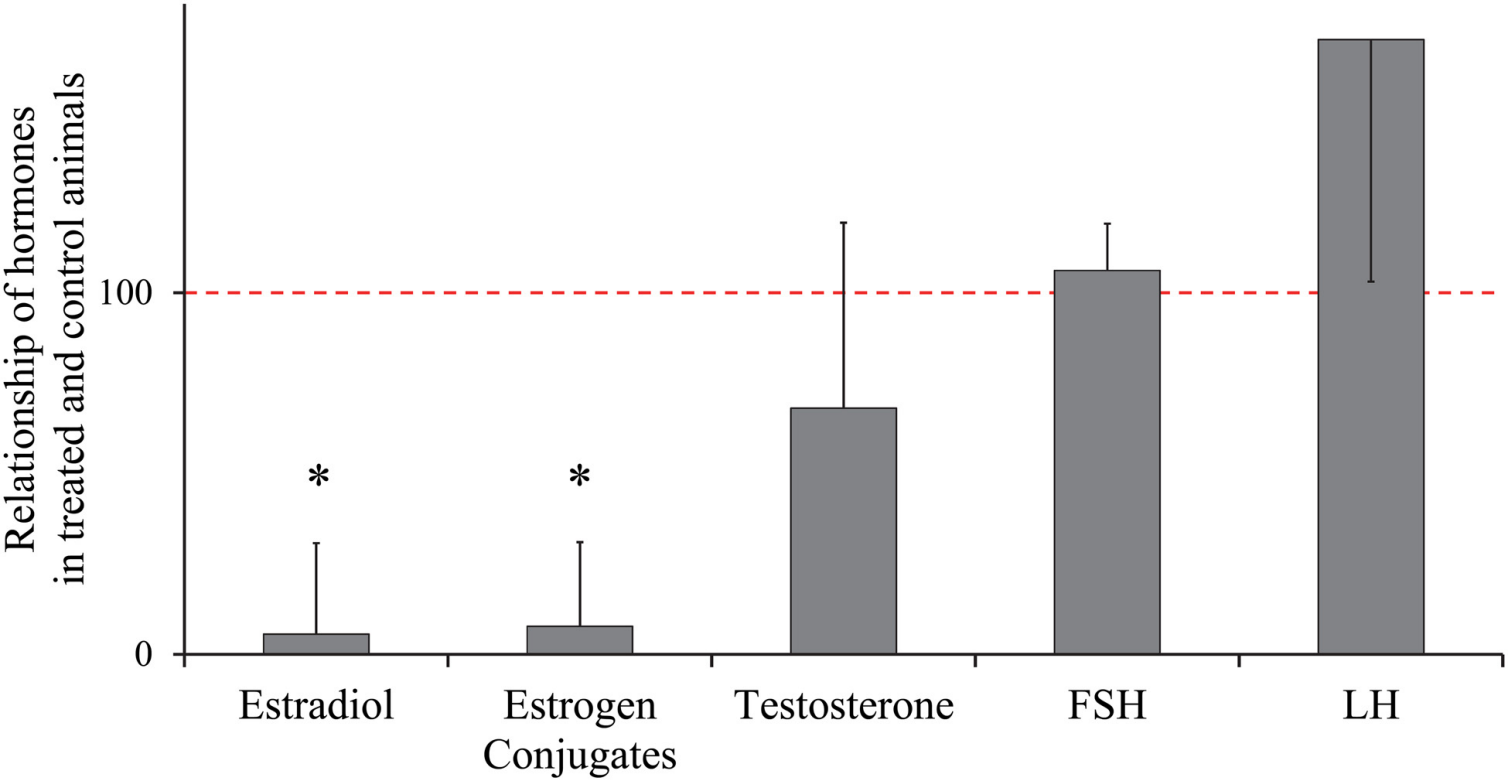
Hormonal response to letrozole treatment. Hormone levels in control animals were normalized to 100, indicated by the dotted line. Estradiol, estrogen conjugates and testosterone are testicular tissue levels. FSH and LH are plasma levels. * indicates significant difference from control levels (p < 0.05) [24].

Five additional littermates to the 16, 20 and 40 week old animals described above were treated weekly with an oral dose of 0.1 mg/kg letrozole (CGS 20267; 4-40-(1H-1,2,4-triazol-1-yl-methylene)-bis-benzonitrile; Ciba-Geigy, Basel, Switzerland), a non-steroidal aromatase inhibitor, from 11 to 16 weeks of age. This treatment effectively reduces both testicular and serum estrogen in the boar including these specific boars without affecting FSH, LH or testosterone (Fig 1) [24,25,29]. Tissue was collected at 16 weeks from the treated littermates concurrently with the vehicle controls described above. Body weight of these boars were 67.1 kg, SEM = 3.9.

### Tissue Collection and Processing

Epididymides were dissected free of the testis and connective tissue, separated into caput, corpus and cauda regions and 4 mm pieces were immersed in 4% paraformaldhyde for 24 hours at 4°C. Tissues were then dehydrated and processed in a VIP Tissue Tech processor (Sakura Finetek USA, Torrance, CA) followed by paraffin embedding. Blocks were sectioned at 5 μm thickness and stained with hematoxylin and eosin. Morphological analysis was performed during visualization with an Olympus BH2 microscope.

### Incidence of Apical Blebbing

The incidence of apical blebbing has not previously been quantified in an organ. Therefore, two methods for quantification of apical blebbing within anatomical regions of the epididymis were assessed. Initial evaluation examined the proportion of longitudinal regions positive for apical blebbing; this evaluation was not readily reproducible without massive sampling. The second method evaluated the percentage of tubule cross sections with apical blebbing in at least four tissue sections per region per animal. These four tissue sections had an average of 1,258 and 772 tubule cross sections which were evaluated in caput and corpus regions, respectively. Tissue sections were separated by at least 25 μm in the paraffin block to verify that positive apical blebbing events were not duplicates. The single reader was blinded to treatment. Coefficients of variation were calculated for groups of up to ten tissue sections from each region and each age group to determine the number of tissue sections required for reproducible results. The number of tissue sections to be used was selected based upon an upper limit for coefficient of variation of 10%, (Table 1) with the exception of the 11 week caput data, which included many zero values.

**Table 1:** Coefficient of variation for apical blebbing as a proportion of the total tubule number.

	Age (weeks)			
Region	11	16	20	40
Caput	19.75	7.95	2.34	0.75
Corpus	8.23	0.73	0.19	2.04

Calculated from four tissue sections per animal per age per region as the percentage of round tubules positive for blebbing.

### Evaluation of Apoptosis

To evaluate incidence of programmed cell death, TUNEL assay was performed on a single animal from each age group. Briefly, 5 μm paraffin tissue sections were rehydrated, subjected to proteinase K antigen retrieval, incubated with terminal deoxynuceotidyl transferase (TdT enzyme) which enzymatically labeled the free 3’-OH termini with digoxigenin-conjugated nucleotides, followed by incubation with the antibody to digoxigenin-conjugated nucleotide and visualized using 3,3’-diaminobenzidine (DAB), according to manufacturer’s instructions (Apoptag, Millipore, Billerica, MA). Negative controls were exposed to all of the steps except the TdT enzyme. Following visualization of slides labeled with TUNEL, the same slides were stained with hematoxylin and eosin for visualization of blebbing cells.

### Association of Clear Cells with Apical Blebs

Labeling of V-ATPase, a clear cell marker, was performed on tissues from animals from the 16 and 20 week age groups [30]. Primary antibody (Breton Laboratory, Boston, MA) was diluted to 1:100 and counter stained with 4′, 6-diamidino-2-phenylindole dihydrochloride (DAPI). Slides were visualized with fluorescent microscopy followed by morphological evaluation after staining the same slides with hematoxylin and eosin.

### Statistical Analysis

Data were subjected to analysis of variance (ANOVA) using SAS statistical programs (Proc Mixed, SAS version 9.3, SAS Institute, Cary, NC, USA). A cubed transformation was used to improve normality and the non-transformed data is graphed. A linear regression analysis was performed on non-transformed data with age as a continuous variable. In all cases, a p-value ≤ 0.05 was considered to have statistical significance.

## Results

**Fig 2:**
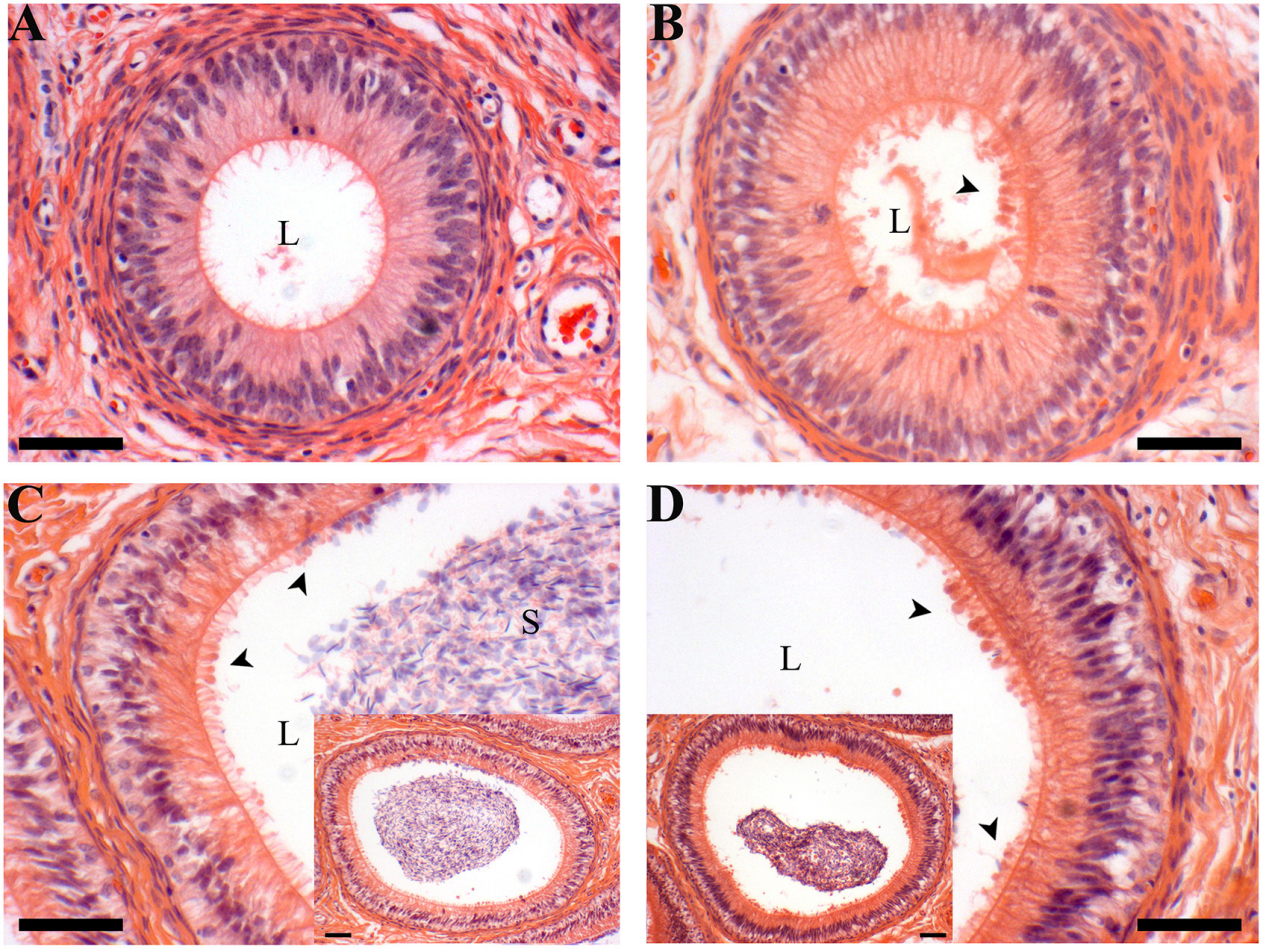
Apical blebbing morphology. **A** 11 week corpus tubule, **B** 16 week corpus tubule, **C** 20 week corpus, inset is lower magnification of same tubule, **D** 40 week corpus, inset is lower magnification of same tubule. Note apical blebbing is evident in both the 20 and 40 week samples (arrowheads); 16 week samples have fewer blebs and this 11 week corpus section has none. L = lumen, S = sperm, scale bars are 5 μm.

**Fig 3:**
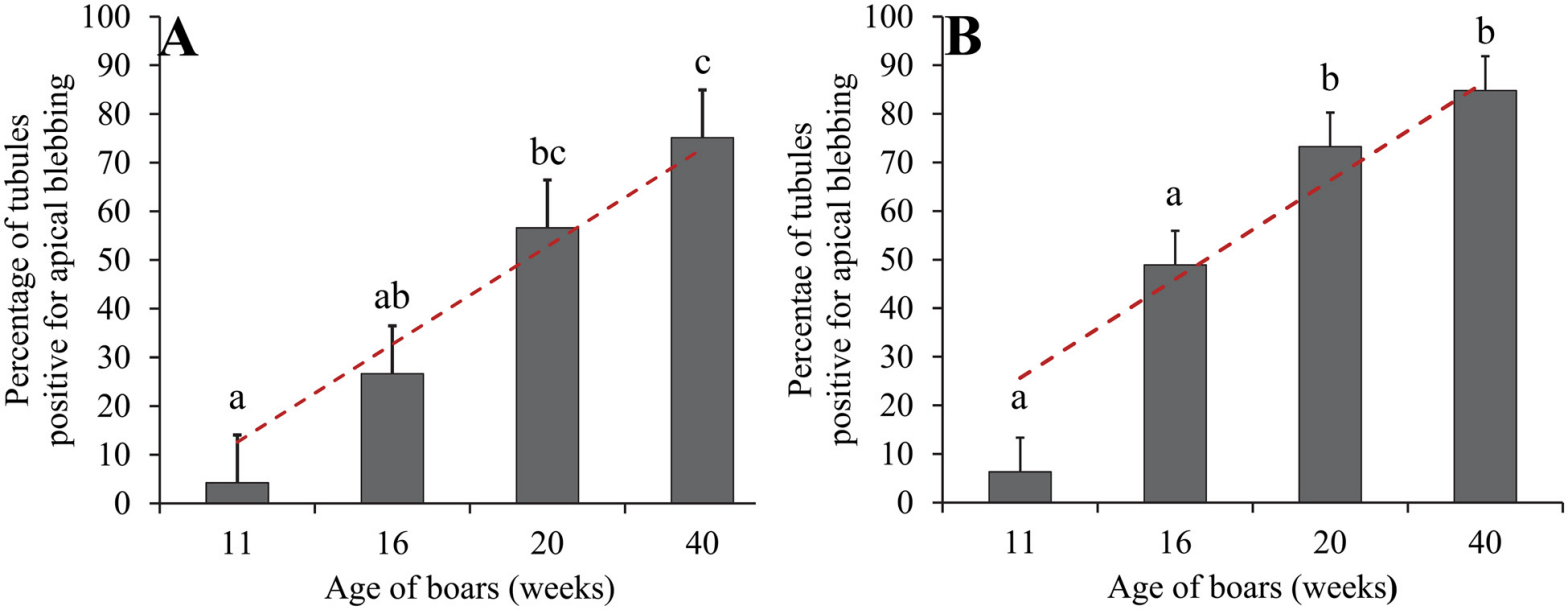
Development of apical blebbing in the caput epididymis (A) and the corpus epididymis (B). Bars represent least squares means and SEM from 5 animals. Values with different superscripts are different in the analysis of transformed data (p ≤ 0.05). The dashed line represents the linear regression with non-transformed data (p < 0.001).

**Fig 4:**
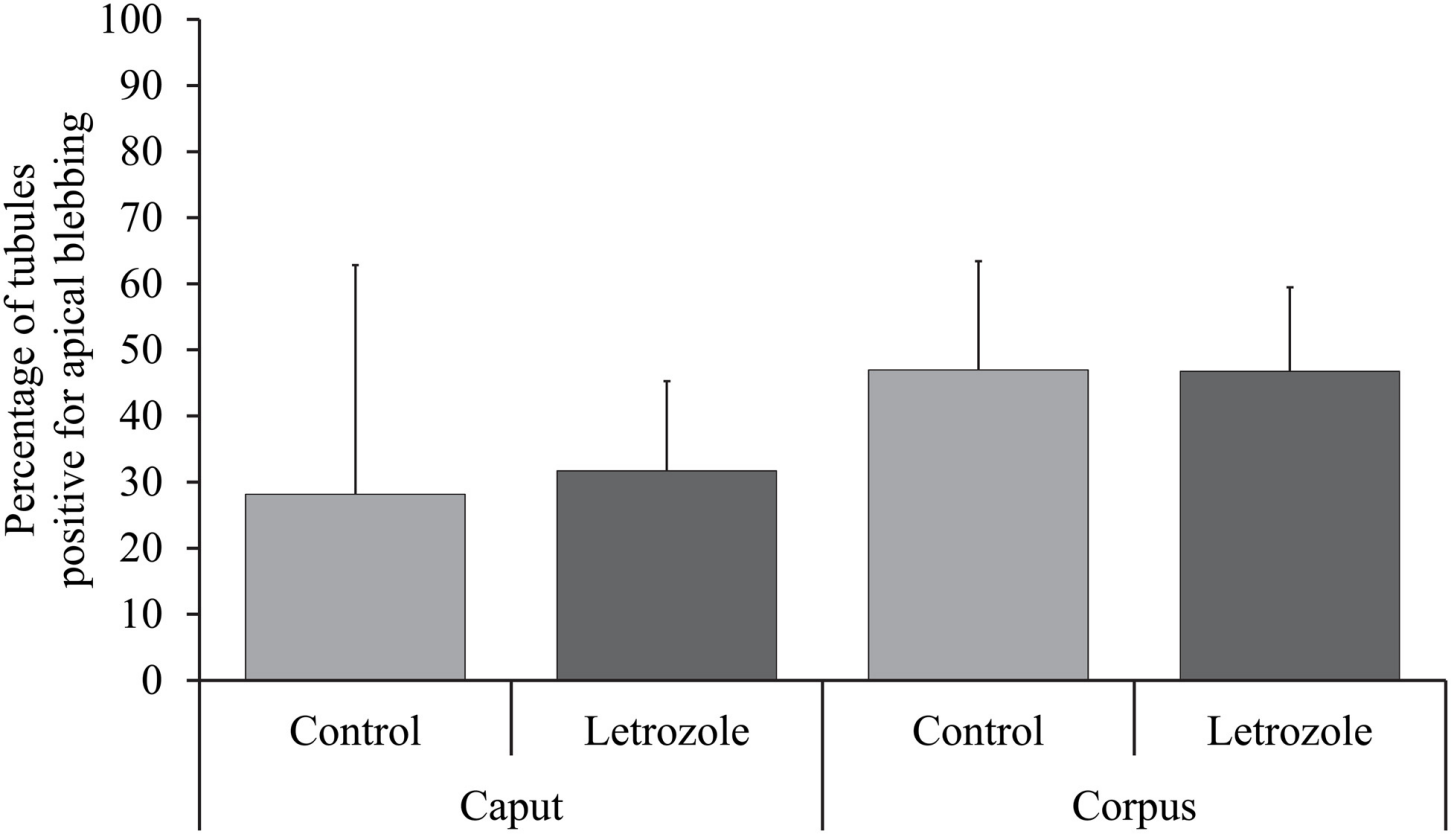
Apical blebbing in letrozole treated versus control littermates. Bars represent least squares means with SEM from 4 and 5 animals in the caput and corpus epidiymis, respectively. Apical blebbing in caput and corpus epididymis were not altered when estrogens were reduced.

Temporal Development and Estrogenic Influence: At 11 weeks of age, very few caput and corpus epididymal tubule cross-sections were blebbing (present in one of the five animals). The incidence increased at 16 weeks of age, which coincided with the onset of puberty in these animals, confirmed by presence of sperm in the epididymal lumen. At this time point, (and at later time points) the apical blebbing rates in the corpus is numerically higher but not statistically different than the rate in the caput. By 20 weeks of age, approximately half of the cross-sections in the caput and three quarters of the corpus cross-sections had blebs. Apical blebbing increased with age in a linear manner (Figs 2 and 3). Letrozole treatment (reduced estradiol) did not affect blebbing in either the caput or the corpus region (Fig 4).

### Apical Blebbing Occurs in Viable Cells

**Fig 5:**
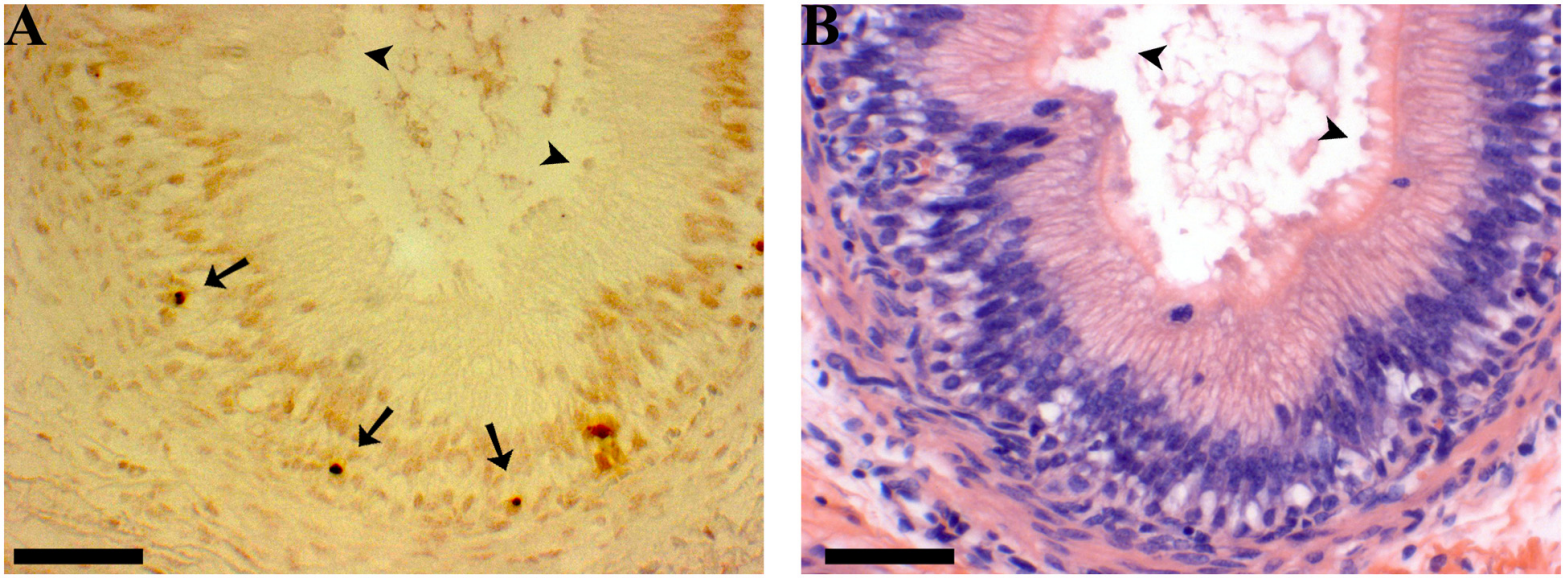
TUNEL labeling of corpus epididymis. **A** Apoptotic cells are dark brown and visible near the basement membrane of the epithelium in 16 week corpus epididymis (arrows). **B** Hematoxylin and eosin stain of the same region as A; apical blebbing is evident in a large segment of the tubule (arrowheads). The apoptotic cells are not numerous enough to account for the apical blebbing seen in B. Scale bars indicate 5 μm.

Apoptosis is occurring at a low rate in the epithelial cells of all age groups in the study. TUNEL positive nuclei were generally close to the basement membrane, below the level of principal cell nuclei (Fig 5). Positive nuclei do not correspond to cells with apical blebbing visualized in the sections subsequently stained with hematoxylin and eosin.

### Clear Cell Relationship to Cells with Apical Blebbing

**Fig 6:**
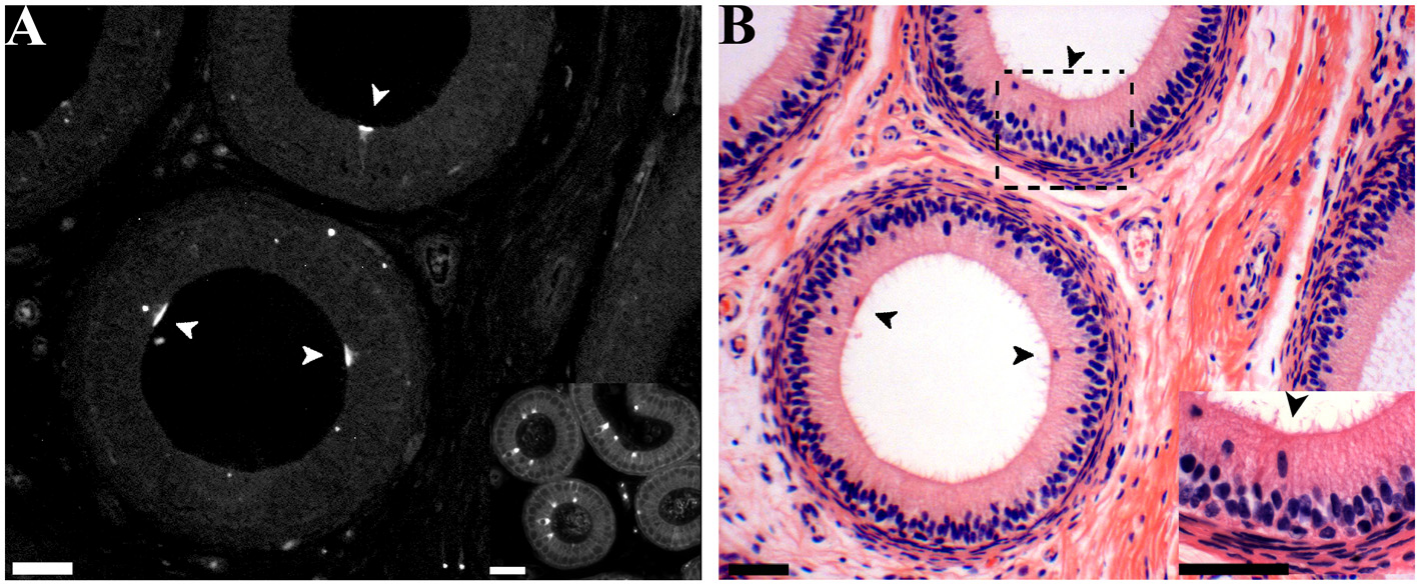
Labeling of clear cells in the caput epididymis. **A** Cross section of caput epididymis from 16 week boar showing V-ATPase positive cells (arrowheads), L = Lumen. Some background staining is evident. Inset: Positive control mouse caput epididymis with similar staining pattern for V-ATPase positive cells. **B** Hematoxylin and eosin staining of the same region as A; arrowheads indicate clear cells. Inset: Higher magnification of boxed area including a clear cell. Scale bars indicate 5 μm.

Immunohistochemical labeling in the pig caput and corpus epididymis identified clear cells with a similar V-ATPase localization to that seen in the mouse (Fig 6). These cells represent a small proportion of the cell population as a whole. These particular clear cells do not demonstrate apical blebbing, although other clear cells may demonstrate apical blebbing. Their small contribution to the cell population in the porcine epididymis does not account for the degree of apical blebbing observed.

## Discussion

Cellular budding is observed in multiple cell types under many conditions. These include cell migration, mitosis, apoptosis and secretion. The appearance of apical blebbing in the porcine epididymis under the conditions examined here does not indicate the final fate of the bleb—they may reabsorb or detach. However, principal cells are not motile, nor are there high rates of mitosis after differentiation. [31,32] It is logical, therefore, to assume that the major function of apical blebbing observed in principal cells of the epididymis is part of a secretion mechanism previously described [12,33–36].

Boars are pubertal at approximately 4 months of age [11]. Steroid hormone production increases and the first sperm reach the lumen of the epididymis at this time. Tight junctions form between principal cells of the epididymis between 6 and 8 weeks of age, indicating a switch in at least some of the cell population toward differentiation versus proliferation [37]. Our preliminary work revealed apical blebbing is not apparent in animals younger than 11 weeks, which directed our focus to development after 11 weeks of age. These results show a linear increase in apical blebbing during development, which supports the hypothesis that apical blebbing is a mature phenotype. This is true in both the caput and corpus regions of the epididymis, although the rate of blebbing is numerically lower in the caput.

The regions of the epididymis experience different regulatory environments, thus caput and corpus regions were analyzed separately [13,38]. The cauda was excluded from the analysis due to the nature of the tubule in this region. A major function of the cauda epididymis is to store spermatozoa. The tubule is much larger in diameter and more difficult to adequately evaluate using the parameters established in the caput and corpus regions.

To verify that the observed apical blebbing was part of a secretion process rather than the result of programmed cell death, apoptosis was assessed. Low levels of apoptotic cells were apparent in the corpus epithelium from each age group. Comparisons of these results with the high incidence of apical blebbing occurring throughout the tissue indicate that apoptotic cells cannot be the primary source of membrane blebs. The TUNEL positive nuclei were present near the basement membrane, away from the majority of the nuclei in the epithelium, i.e., principal cells, the primary blebbing cell [35]. Therefore, apical blebbing appears to be part of the normal secretion of viable cells rather than cells undergoing apoptosis, although a low level of blebbing events due to apoptosis would be included in the data. Moreover, our data collection does not count individual events of blebbing; rather the data collected is what proportion of the epididymal tubule is undergoing blebbing at the time of sample collection. A single blebbing cell, consistent with a positively stained apoptotic cell, was not observed. Our observation of apoptosis levels confirms that the vast majority of the blebbing found in our samples is attributable to normal cellular function. Clear cells are the second most common cell type to reach the lumen of the epididymis and are known to bleb [39], therefore we assessed their potential contribution to apical blebbing in the porcine epididymis using V-ATPase as a marker [40]. A very low proportion of the porcine epididymal epithelial population is clear cells, which further verifies that apical blebbing is primarily derived from principal cells.

Having established the age at which apical blebbing appears, we next evaluated the role of estrogen in regulating apical blebbing. Estrogen is a major regulatory factor in male reproduction. Apical blebbing is upregulated at the same time that the boar is increasing production of steroid hormones. Estrogen appears to be involved in bleb formation in the rat coagulation gland [23,41]. Therefore, a role for estrogen in apical blebbing in the epididymis was our next hypothesis. Letrozole, an aromatase inhibitor, is very effective in blocking aromatase conversion of androgens to estrogens in the boar [24,25]. This model is ideal to evaluate the role of estrogen in apical blebbing as testosterone and gonadotropins are not altered by letrozole treatment in the boar, including these animals [24]. We found the rate of apical blebbing was unaltered by treatment with letrozole compared with littermate controls. Therefore, estrogens do not appear to either up regulate or down regulate apical blebbing in the epididymis. Perhaps the difference in regulation of blebbing in the epididymis versus the coagulation gland is due to the differing developmental origins of these two organs or to species differences.

In conclusion, apical blebbing appears to be a mature phenotype in the epididymis. The onset of apical blebbing occurs during puberty and the rate increases in a linear manner through 40 weeks of age, sexual maturity in our model. The majority of apical blebbing in the epididymis is not attributable to the clear cell population, although other cell types may make minor contributions. Nor is it due to apoptosis; instead apical blebbing is a secretion mechanism attributable to the principal cell population. Apical blebbing appearance is not influenced by estrogens, a major hormonal regulator in the epididymis. However, a number of other hormonal and/or luminal factors (e.g. androgens, spermatozoa) may regulate this energetically demanding secretion process.
